# Hypoxia and connectivity in the developing vertebrate nervous system

**DOI:** 10.1242/dmm.037127

**Published:** 2018-12-12

**Authors:** Joshua L. Bonkowsky, Jong-Hyun Son

**Affiliations:** 1 Department of Pediatrics, University of Utah, Salt Lake City, UT 84108, USA; 2 Department of Biology, University of Scranton, Scranton, PA 18510, USA

**Keywords:** Connectivity, Hypoxia, Neuroscience, Pathfinding

## Abstract

The developing nervous system depends upon precise regulation of oxygen levels. Hypoxia, the condition of low oxygen concentration, can interrupt developmental sequences and cause a range of molecular, cellular and neuronal changes and injuries. The roles and effects of hypoxia on the central nervous system (CNS) are poorly characterized, even though hypoxia is simultaneously a normal component of development, a potentially abnormal environmental stressor in some settings, and a clinically important complication, for example of prematurity. Work over the past decade has revealed that hypoxia causes specific disruptions in the development of CNS connectivity, altering axon pathfinding and synapse development. The goals of this article are to review hypoxia's effects on the development of CNS connectivity, including its genetic and molecular mediators, and the changes it causes in CNS circuitry and function due to regulated as well as unintended mechanisms. The transcription factor HIF1α is the central mediator of the CNS response to hypoxia (as it is elsewhere in the body), but hypoxia also causes a dysregulation of gene expression. Animals appear to have evolved genetic and molecular responses to hypoxia that result in functional behavioral alterations to adapt to the changes in oxygen concentration during CNS development. Understanding the molecular pathways underlying both the normal and abnormal effects of hypoxia on CNS connectivity may reveal novel insights into common neurodevelopmental disorders. In addition, this Review explores the current gaps in knowledge, and suggests important areas for future studies.

## Abstract (precis)

**Summary:** The nervous system's exposure to hypoxia has developmental and clinical relevance. In this Review, the authors discuss the effects of hypoxia on the development of the CNS, and its long-term behavioral and neurodevelopmental consequences.

## Introduction

The developing nervous system depends upon precise regulation of oxygen levels. Anoxia, the lack of oxygen, or hypoxia, the condition of low oxygen concentration (Boxes 1 and 2), can interrupt developmental sequences and cause a range of molecular, cellular and neuronal changes and injuries. Hypoxia plays a normal physiological role during the development of the vertebrate embryo, including promoting the use of anaerobic metabolism, driving vasculature formation, supporting the development of the heart and bones, and stimulating the migration of neural crest cells (Dunwoodie, 2009). However, non-physiological hypoxia disrupts embryonic development and can lead to death.

**Box 1. Measuring oxygen levels**

Oxygen levels are indicated in the atmosphere as percent oxygen (21% in the Earth's atmosphere) or the partial pressure of oxygen (pO_2_). In water, oxygen content reaches saturated equilibrium with the atmosphere and results in an oxygen content of 9 mg/l. Oxygen levels in water can be measured directly with an oxygen meter. In a cell or organ, such as a neuron or the brain, oxygen levels are affected by additional considerations, including oxygen capacity of the blood, cardiac output, oxygen delivery in the lungs, etc. However, in a small animal such as *C. elegans* or the embryonic zebrafish, oxygen delivery is determined by diffusion.

An absence or decrease of oxygen (e.g. hypoxia) in tissues and cells can be detected and in some instances quantified by a variety of methods. These include the use of a chemical-based dye probe, for example pimonidazole hydrochloride, which binds macromolecules in hypoxic tissue (Raleigh et al., 1987), or of a genetic indicator of hypoxia pathway activation such as a zebrafish transgenic prolyl hydroxylase (PHD) GFP reporter (Santhakumar et al., 2012). Specialized magnetic resonance imaging (MRI) techniques offer a radiological measure (O'Connor et al., 2018). Additionally, researchers can use a tissue sensor, for example near-infrared spectroscopy (NIRS), to measure tissue oxygen saturation (Liao and Culver, 2014).

**Box 2. Glossary**

**Anisotropy:** a measure of water diffusion. For magnetic resonance imaging (MRI)-based neuroimaging, the fractional anisotropy provides a measure of water diffusion in three dimensions that can report on fiber density, axon diameter and myelination in white matter.

**Anoxia:** the complete absence of oxygen or oxygen delivery.

**Connectivity development:** the processes in the CNS concerned with the development of connections and circuitry. In particular, the aspects of axon elongation and pathfinding, and of synapse formation, stabilization and pruning.

**Diffusion tensor imaging (DTI):** an MRI-based neuroimaging technique that can determine the location, orientation and anisotropy of the brain's white matter tracks.

**Dopaminergic diencephalospinal tract (DDT):** a descending axon track from dopaminergic neurons in the diencephalon to targets in the spinal cord.

**Eph receptors:** a family of cell-surface receptor tyrosine kinases involved in axon pathfinding as well as other biological processes. Their ligands are the ephrins.

**Hypoxia:** a decrease in oxygen levels or content. Hypoxia is determined by comparison to ‘normal’ or physiological levels. For example, normal oxygen levels in the adult human brain range from 0.5 to 8% (Dings et al., 1998).

**Hypoxia-ischemia:** a combination of hypoxia with decreased or absent blood flow.

**NMDA receptor:** N-methyl-D-aspartate (NMDA) receptor; a multi-subunit cell-surface receptor for glutamate involved in synaptic plasticity in mature animals and in axon pathfinding during development.

**Partial pressure of oxygen (pO_2_):** the amount of oxygen in a liquid or in the atmosphere.

Understanding hypoxia's effects has important public health considerations because hypoxia is a major complication of prematurity. Every year, almost 400,000 infants in the US and 15 million infants globally are born prematurely (defined as a birth prior to 37 weeks gestation) (Martin et al., 2011b; Beck et al., 2010). While the prematurity-associated effects and pathophysiology of anoxia or hypoxia-ischemia (Box 2) on the developing brain are well described and have been extensively studied (du Plessis and Volpe, 2002; Ferriero, 2004; Volpe, 2009; Back, 2014), hypoxia's effects are less well understood.

Work over the past decade has revealed that hypoxia causes specific disruptions in the development of central nervous system (CNS) connectivity, altering axon pathfinding and synapse development (Box 2). Alterations in neuronal connectivity are associated with neurodevelopmental disorders (NDDs) ranging from autism spectrum disorders (ASDs) to intellectual impairment (Geschwind and Levitt, 2007; Baribeau and Anagnostou, 2013; Shepherd, 2013; Lee et al., 2018). Rates of NDDs are elevated in children who were born prematurely (approaching 35% of premature children) and are correlated with exposure to hypoxia (Horwood et al., 1998; Vohr et al., 2000; Bass et al., 2004; Firme et al., 2005; Barrett et al., 2007; Saigal and Doyle, 2008; Johnson and Marlow, 2011; Martin et al., 2011a).

The goals of this article are to review hypoxia's effects on the development of CNS connectivity, including the molecular mediators and the changes in gene expression, CNS circuitry and function due to regulated as well as inadvertent mechanisms. Animals appear to have evolved genetic and molecular responses to hypoxia during CNS development that result in functional behavior alterations. Understanding the molecular pathways involved in both the normal and abnormal effects of hypoxia on CNS connectivity may reveal novel insights into common neurodevelopmental disorders. In addition, this Review explores the gaps in our knowledge, and suggests important areas for future studies.

## Basic molecular elements of the hypoxia response

**Fig. 1.**
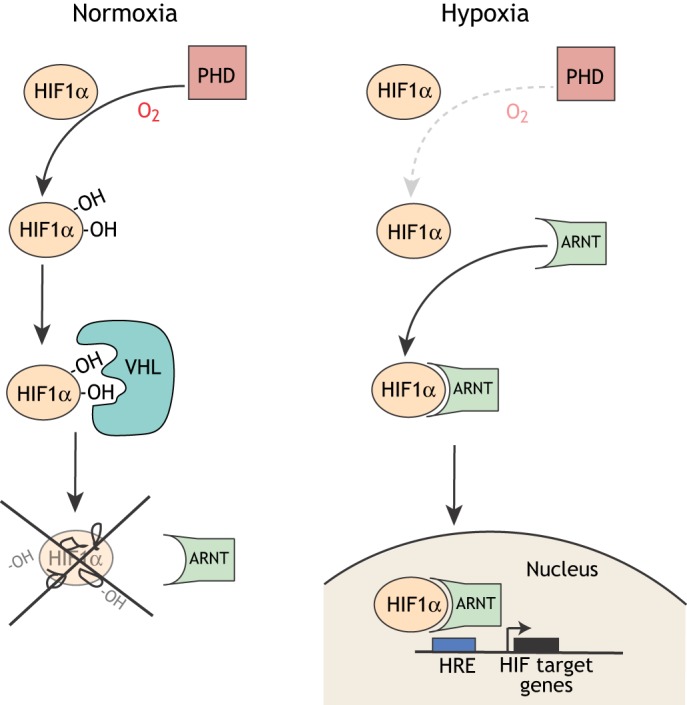
**A simplified schematic of the basic cellular response to hypoxia.** In normoxia, prolyl hydroxylases (PHDs) hydroxylate HIF1α, which allows the Von Hippel Lindau (VHL) protein to bind HIF1α, leading to degradation of HIF1α. Under hypoxic conditions, HIF1α is not hydroxylated or degraded and dimerizes with ARNT (also known as HIF1β), translocates to the nucleus and, with the transcriptional coactivator CBP/p300, binds genomic DNA at hypoxia response elements (HREs) to activate transcription of target genes.

The vertebrate response to hypoxia is complex, but has central components that are well understood. Chiefly, the response is controlled by transcription factors, the hypoxia-inducible factors (HIFs), and by cellular oxygen sensors, the prolyl hydroxylase domain proteins (PHDs) (Fig. 1) (Semenza, 2007). HIF is a heterodimer of HIF1α and HIF1β [the latter is also known as aryl hydrocarbon receptor nuclear translocator (ARNT)]. In mammals, ARNT is constitutively expressed, but HIF1α is regulated by oxygen concentration via PHDs. In normoxic conditions, PHDs hydroxylate HIF1α, which allows binding of the von Hippel-Lindau tumor suppressor protein (pVHL) and leads to subsequent ubiquitylation and proteasomal degradation of HIF1α (Ruas and Poellinger, 2005; Schofield and Ratcliffe, 2005). In hypoxic conditions, hydroxylation is reduced, HIF1α accumulates, dimerizes with ARNT, and translocates to the nucleus to activate the transcription of hundreds of genes (Sharp and Bernaudin, 2004).

HIF1α is the central mediator of the CNS response to normal hypoxia exposure during development (as it is elsewhere in the body). However, abnormal severity and/or timing of hypoxia and HIF1α expression causes a dysregulation of gene expression (Box 3). But what is different in hypoxic injury compared to normal CNS development? This dichotomy for the role of HIF1α raises questions on the identity, roles and mechanisms of the key mediators of HIF1α’s effects on CNS connectivity development (Box 2) from hypoxic injury.

**Box 3. Genes and signaling pathways controlling connectivity and their regulation by hypoxia**

Development of CNS connectivity is a critical step for brain development that requires a precisely orchestrated process of axon guidance and pathfinding, synapse development and stabilizations. Our laboratory as well as others have shown that hypoxia disrupts CNS connectivity (Pocock and Hobert, 2008; Stevenson et al., 2012). To further characterize changes in the expression levels of genes related to CNS connectivity by hypoxia, we performed a transcriptional profiling study in zebrafish, examining the response of a subset of 1270 genes selected for their roles in the development of CNS connectivity. We found that hypoxia disproportionately affects a subset of connectivity genes, altering both the levels and the timing of expression during embryonic development (Milash et al., 2016). For example, the receptor/ligand pair *plxnA3* and *sema3ab* are both upregulated by hypoxia, which could lead to elevated GTPase activity and increased repulsive axon guidance (Palaisa and Granato, 2007). However, it is still not clear why specific genes are more vulnerable to the effects of hypoxia and what the overall effects those gene changes have on connectivity in the developing CNS.

Interestingly, while experimental work (loss-of-function and mis-expression experiments in zebrafish) demonstrated that *hif1a* mediates connectivity disruption, the analysis of the changes in mRNA levels of different HIF isoforms showed only relatively minor changes in response to hypoxia, similar to a previous report in zebrafish (Rytkönen et al., 2014). This suggests that regulation of HIF isoform activity by hypoxia is predominantly occurring at the post-transcriptional stage (Yee Koh et al., 2008).

## Role of other hypoxia-response factors and other mechanisms

Although much of the molecular response to hypoxia is regulated by HIF1α, there are other hypoxia-response transcription factors, such as HIF2 and HIF3, that also regulate gene expression in response to changes in oxygen tension (Majmundar et al., 2010; Wang and Semenza, 1993). HIF1 and HIF2 largely function as transcriptional activators. Their target genes partially overlap with those of HIF1, but they also control the expression of unique target genes. However, HIF1 appears to be mainly responsible for the initial adaptation to hypoxia, whereas HIF2 expression begins after more prolonged oxygen depletion (Koh and Powis, 2012; Bartoszewska et al., 2015).

More recent studies demonstrate further complexity of the genetic regulatory response to hypoxia by HIF3α (Janaszak-Jasiecka et al., 2016). *HIF3A* has a large number of mRNA splice variants, and displays a dual role in response to hypoxia: it suppresses HIF1- and HIF2-mediated gene expression, but it also induces the expression of a specific subset of target genes by binding to a hypoxia-response element (HRE). The HIF3α HRE is distinct from the canonical HIF1α HRE (Ravenna et al., 2016). Finally, the effects of hypoxia on connectivity can also be indirectly mediated. For example, *in vitro* studies of cultured neurons show that hypoxia reduces synaptic activity, a known controller of synapse development, and also reduces overall network connectivity (Fujiwara et al., 1987; Hofmeijer et al., 2014). Hypoxia can also affect the overall health and homeostasis of neurons, as shown by changes in acid-base transporters in the CNS of juvenile mice exposed to hypoxia (Douglas et al., 2003).

## The contribution of hypoxic injury to human neurodevelopmental disorders and abnormal CNS development

Hypoxic injury is a clinical mechanism that is distinct from anoxia or hypoxia-ischemia (Box 2), although there is a continuum of effects and of pathophysiology. Premature infants are the population at greatest risk for chronic hypoxic injury and for the subsequent adverse neurocognitive and neuropsychiatric outcomes (Horwood et al., 1998; Johnson and Marlow, 2011). Premature infants can experience up to 600 hypoxic episodes per week, each lasting at least 10 s or more (Martin et al., 2011a). The reason(s) for this chronic hypoxia are not well understood. Abnormal autonomic regulation, particularly of the cardiopulmonary system, may be a likely primary cause, but etiologies can also include placental insufficiency, lung disease, pulmonary hypertension or congenital heart disease. The converse condition, hyperoxia, in which oxygen concentrations are elevated by medical interventions such as those often performed for premature infants, also disrupts normal brain development (Deuber and Terhaar, 2011; Reich et al., 2016).

The hypoxic exposure of premature infants occurs from approximately 12 weeks post-conception age (PCA) through term birth (40 weeks PCA) (Martin et al., 2011a, 2012). This developmental window overlaps with the timing of premature births from 24-36 weeks PCA, and includes the time when axon and synaptic connections are forming in the human CNS (ten Donkelaar et al., 2004; Ren et al., 2006; Kostović and Jovanov-Milosević, 2006; Molyneaux et al., 2007; Stiles and Jernigan, 2010; Vasung et al., 2010; Semple et al., 2013). Changes in synapse development and function have been recognized as a significant component of NDDs caused by prematurity and hypoxia (Gilman et al., 2011).

Exposure to hypoxia is associated with worse outcomes in premature babies. In the long term, up to 35% of premature infants will develop an NDD, such as attention-deficit disorder, autism, cerebral palsy, motor impairment, depression, epilepsy or intellectual disability (Bass et al., 2004; Barrett et al., 2007; Laursen et al., 2007; Saigal and Doyle, 2008; Williams et al., 2010; Salmaso et al., 2014). Ironically, while survival rates for premature infants have improved dramatically and the total number of ex-premature infants (an infant or child who was born prematurely but is now older) has increased over the past decade (Mathews et al., 2011), neurodevelopmental outcomes have not improved and therapeutic strategies are lacking (Fanaroff et al., 2007; Hintz et al., 2011). This has resulted in an increased number of children with NDDs. Excess annual costs related to premature birth are US$26.2 billion (McCabe et al., 2014). Indeed, the lack of understanding of the fundamental mechanisms contributing to the development of NDDs from prematurity and hypoxia has limited the development of therapies.

## Effects of hypoxia on connectivity development

The first data clearly demonstrating the effects of hypoxia on neuronal connectivity were collected in *Caenorhabditis elegans*. An elegant paper by the Hobert group showed that hypoxia specifically disrupted axon pathfinding through a *hif1a-*dependent mechanism by upregulating the Eph receptor (Box 2) VAB-1 (Pocock and Hobert, 2008). In a subsequent paper, the same group showed that hypoxia led to the use of a latent neuronal circuit, suggesting that exposure to hypoxia had altered the connectivity of a sensory pathway (Pocock and Hobert, 2010).

**Fig. 2.**
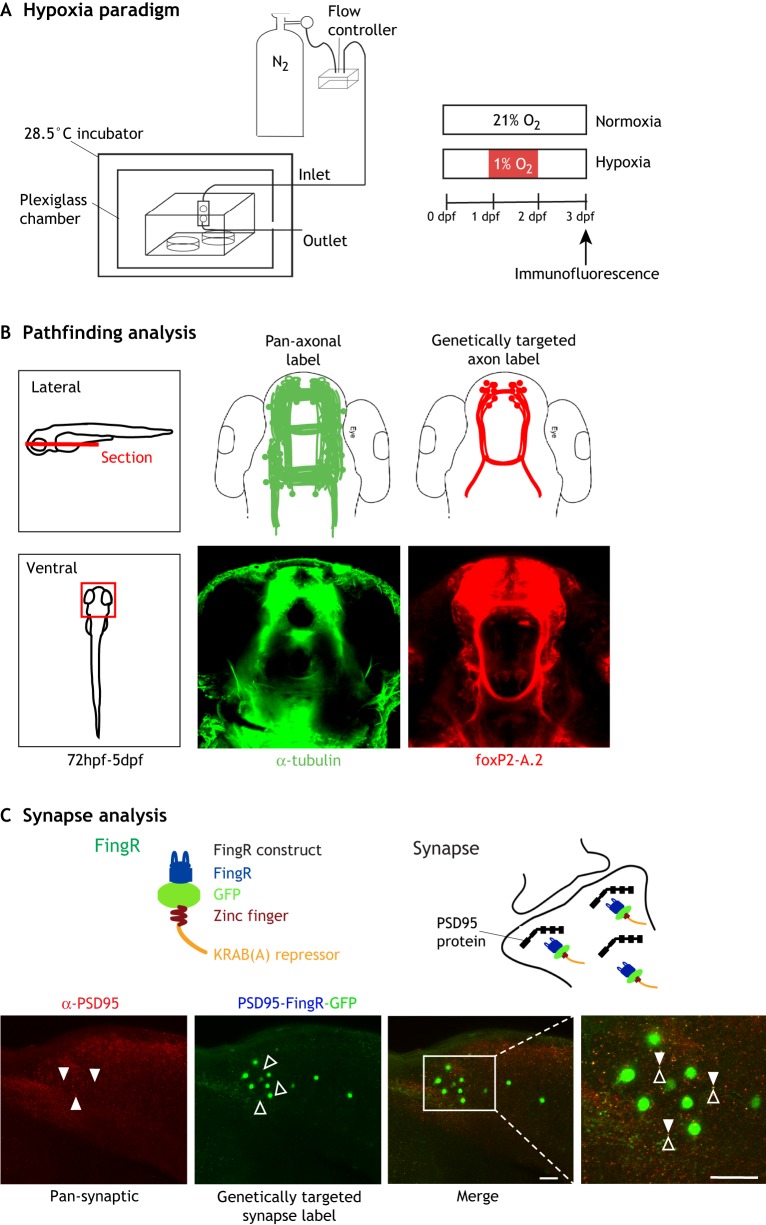
**The hypoxia experimental system and examples of methods for the analysis of CNS connectivity.** (A) Schematic diagram of the hypoxia system for experiments with zebrafish (Stevenson et al., 2012). Embryos are placed in a sealed plexiglass chamber where oxygen levels are manipulated by a controller that monitors oxygen (O_2_) levels and adjusts nitrogen (N_2_) flow to displace O_2_. Altering the timing of hypoxia exposure allows researchers to examine the effects on connectivity at different stages of embryo development, for example, via immunofluorescence axon labeling (experimental procedure shown on the right). (B) Examples of axon pathfinding analysis. The left panel shows the region of the zebrafish CNS imaged in the analysis. The center and right panels show confocal images of the forebrain, both of which are dorsal views with the rostrum at the top. A pan-axonal antibody such as anti-(acetylated) tubulin (central panel, green) labels all of the axon tracts, which permits visualization of significant changes in axon pathfinding upon exposure to hypoxia. An axon reporter expressed in a genetically defined group of axon tracts, for example EGFP-CAAX driven by the *foxP2-A.2* enhancer (right panel, red) (Bonkowsky et al., 2008), only labels a subset of axons but allows more precise tracking of axon pathfinding changes. (C) Examples of synapse analysis. Confocal images show lateral views of the zebrafish trunk/spinal cord, dorsal to the top, rostral to the right. A pan-synaptic antibody (anti-PSD95; red signal, white arrowheads) labels all synapses; this makes it difficult to determine what is happening to any specific set of neurons. A genetically targeted synapse label, such as a FingR (Son et al., 2016), shown schematically, can be targeted against PSD95. By expressing the PSD95-FingR (green signal, black arrowheads) under the control of an enhancer or other transgene, researchers can track hypoxia-induced synaptic changes in a genetically defined group of neurons. Scale bars: 50 µm (10 µm in enlarged image).

The first experiments showing an effect of hypoxia on axon pathfinding in vertebrates were performed using zebrafish embryos as a model system (Fig. 2) (Stevenson et al., 2012). The authors found disrupted CNS connectivity development as demonstrated by a loss of midline axon crossing. This was later confirmed by single-neuron labeling, which showed that axons were making specific pathfinding errors (Xing et al., 2015). The effects on axon pathfinding were specific to hypoxia exposure during a specific developmental time period in zebrafish embryogenesis, and the pathfinding errors were *hif1a* dependent. Interestingly, the pathfinding errors were partially mediated by ephrinB2a, similar to the observation of Eph dependence in *C. elegans* discussed above. Importantly, Xing and colleagues showed that, in their system, exposure to hypoxia did not cause an increase in apoptosis or affect other aspects of neurogenesis or cell fate determination. This was important because it demonstrated that the manner in which hypoxia affected pathfinding was consistent with a direct effect, and was not secondary to the loss of key structural cells, for example. Other hypoxia paradigms can have effects on apoptosis and cell fate determination (e.g. Vangeison et al., 2008; le Feber et al., 2017).

In other vertebrates (including humans, discussed in the next section), magnetic resonance imaging (MRI) metrics have been used to provide indirect measures of connectivity changes due to hypoxia. Changes in diffusion tensor imaging (DTI) and fractional anisotropy (Box 2) have shown that hypoxia alters these measures in mice (Chahboune et al., 2009; Cengiz et al., 2011; Cai et al., 2012) and guinea pigs (Kim et al., 2015).

We do not know whether specific axon tracts and/or neuron types are more susceptible to hypoxia. While certain midline crossing tracts are clearly affected, this may simply reflect that it is experimentally easier to assess the changes in commissural axons compared to longitudinal ones. Also, it is unclear whether the axon tracts in which the Eph/Ephrin family members control pathfinding are particularly responsive to hypoxia (Pocock and Hobert, 2008; Stevenson et al., 2012). However, hypoxia-mediated altered expression of other cell-surface receptors involved in axon pathfinding, including for example *semaphorin3ab* or *dcc*, suggests that this may not be the case (Milash et al., 2016).

The genetic pathway(s) linking hypoxia to changes in axon pathfinding continue to be elucidated. Hif1α activation is shared across species, as is the involvement of the Eph/Ephrin receptor/ligand gene family (Pocock and Hobert, 2008; Stevenson et al., 2012). The neurotransmitter serotonin (5-HT) has also been shown to respond to hypoxia, although with different responses in *C. elegans* (Pocock and Hobert, 2010) compared to vertebrates (Xing et al., 2015). In zebrafish, hypoxia decreases the levels of 5-HT, and 5-HT acts on ephrinB2a expression to regulate axon pathfinding (Xing et al., 2015). It is not clear whether 5-HT's regulation of ephrinB2a is the sole manner by which hypoxia regulates Eph/Ephrin expression. Hypoxia also decreases the expression of the *grin1a* and *grin1b* subunits of the N-methyl D-aspartate (NMDA) receptor (Box 2). The NMDA receptor regulates the midline axon crossing decision through its control of spontaneous neuronal electrical activity, independent of ephrinB2a levels (Gao et al., 2018). It thus appears that hypoxia's effects on axon pathfinding are mediated by several mechanisms.

Researchers have also investigated the effects of hypoxia on synapse development, although experimental data are currently sparse. Rodent gene expression microarray data (Curristin et al., 2002), immunohistochemical studies (Valdez et al., 2007) and zebrafish RNA sequencing (Milash et al., 2016) have demonstrated that hypoxia alters the expression of genes involved in synaptogenesis. The expression of both pre- and post-synaptic genes is altered by hypoxia (Curristin et al., 2002; Milash et al., 2016). Cell culture experiments using mouse hippocampal neurons show that hypoxia causes a reduction in dendritic spine numbers and impairs synaptic activity (Segura et al., 2016), as well as a persistent decline in synapse numbers (Stoyanova et al., 2016; le Feber et al., 2018). However, these studies were performed on neurons from mature animals, so whether the findings can be extrapolated to synaptic changes during embryonal development is unknown.

Direct visualization of synaptic changes has demonstrated that exposure to hypoxia leads to a reduction in the number of synapses between descending dopaminergic axons [the dopaminergic diencephalospinal tract (DDT); Box 2] and spinal cord motor neurons (Son et al., 2016). The mechanism(s) by which hypoxia alters synapse development are unclear. Dopamine signaling and the DDT are necessary for the maturation of locomotion in zebrafish (Lambert et al., 2012). In mammals, the A11 diencephalon nucleus and its axons, the DDT, are also dopaminergic (Koblinger et al., 2014), and the DDT modulates motor behavior through the release of dopamine during locomotion in both mouse and zebrafish (Jay et al., 2015; Sharples et al., 2015). This raises the intriguing possibility that dopamine itself may play an important role in the effects of hypoxia on synapses.

## Human data

Although limited, there are some data regarding CNS connectivity changes from humans who live and/or were born at high altitude, which essentially imposes chronic exposure to hypoxia. Changes in electroencephalogram (EEG) patterns of Bolivian children born at high altitude suggest alterations in neuronal function and/or circuitry (Richardson et al., 2011). Children born and living at higher altitude had a greater likelihood of having an NDD (Wehby, 2013). Interestingly, altered patterns of brain connectivity have been observed even in those individuals who move to a higher altitude as young adults, suggesting the potential for changes in synaptic connectivity (Chen et al., 2017). These limited studies are suggestive, but there is a need for larger epidemiological and imaging studies to evaluate hypoxia-induced CNS connectivity changes and the presence of NDDs. Further, while the genetic and evolutionary changes associated with physiological and hematological responses to high altitude and hypoxia have been studied extensively (reviewed in Azad et al., 2017), there is essentially an absence of any comparable information regarding genetic or evolutionary adaptations of the developing CNS to high altitude and hypoxia.

Another source of information on hypoxia and the potential for changes in human CNS connectivity are studies of human premature births. MRI studies of ex-premature infants who were exposed to chronic hypoxia show alterations in MRI metrics, including changes in functional MRI (fMRI) patterns of activation, suggesting altered connectivity, and reduced fractional anisotropy of white matter tracts, suggesting a decrease in axon tract connections (Gozzo et al., 2009; Mullen et al., 2011; Salmaso et al., 2014). Infants born prematurely have disrupted development of the corpus callosum and other axon tracts of the cerebral hemispheres (Glass et al., 2008; Mullen et al., 2011; de Kieviet et al., 2012; Hasegawa et al., 2011; Thompson et al., 2007; van Pul et al., 2012). Rates of ASDs are three times higher in premature infants (Lampi et al., 2012), and the prevalence of ASDs approaches 25% in the extremely premature, i.e. those born at less than 27 weeks PCA (Limperopoulos et al., 2008). Premature infants who develop ASDs can lack conspicuous brain abnormalities, but can have fMRI changes demonstrating abnormal synchronization of neural activity consistent with alterations in brain connectivity (Dinstein et al., 2011).

## Roles of HIF1α

Although HIF1α mediates many of the responses to abnormal hypoxic exposure, its activity is also necessary for normal development (Dunwoodie, 2009). Physiological hypoxia occurs in the normally developing embryo, including in the vertebrate CNS (Lee et al., 2001; Trollmann and Gassmann, 2009). Further, complete absence of Hif1α leads to embryonic death in mice by embryonic day 11 (E11), and causes CNS abnormalities including neural tube defects and cystic brain abnormalities (Iyer et al., 1998). At this time, no studies have used a conditional knock-out approach to evaluate Hif1α requirements for CNS connectivity. However, early-stage conditional knock-out of *Hif1a* in the CNS of mice has shown that *Hif1a* is necessary for neurogenesis, and that loss of *Hif1a* expression leads to hydrocephalus (Tomita et al., 2003; Wagenführ et al., 2016). An interesting unresolved question is whether the developmental roles of Hif1α are mediated by the presence of physiological hypoxia, or whether this is a hypoxia-independent function. For example, HIF-1 expression is activated by certain bacteria in the gastrointestinal tract (Hartmann et al., 2008). The dichotomy of HIF1α function – its necessity for early CNS development but the adverse effects of its activation by hypoxic injury – has further complicated our understanding of normal versus abnormal functions of HIF1α and its downstream effectors.

## RNAseq and expression data

As discussed previously, in hypoxic conditions, HIF1α is a master regulator of the response to oxygen levels through controlling gene expression. Gene expression profiling studies from *in vivo* and *in vitro* models demonstrated that the activated HIF1α pathway stimulates the expression of a number of genes that promote angiogenesis, energy metabolism and cell survival (Kenneth and Rocha, 2008; Shukla et al., 2018). HIF1α transcriptional targets, including erythropoietin, glucose transporters, glycolytic enzymes and vascular endothelial growth factor, either enhance oxygen delivery to the tissues or facilitate metabolic adaptation to hypoxia (Semenza, 1999; Majmundar et al., 2010).

An *in vivo* analysis of hypoxia's effects on RNA expression changes relative to CNS connectivity development was recently performed in zebrafish. Comparing normoxia to hypoxia, Milash et al. (2016) reported that hypoxia caused a transcriptional desynchronization, both in terms of timing and levels of expression, of genes necessary for connectivity development. These genes (1270 in total) were defined based on their inclusion in the Gene Ontology (GO) terms ‘axon guidance’ or ‘synapse’, and their functions ranged from transcription factors to cell-surface receptors. Interestingly, hypoxia had the most profound effects on the expression of a subset of genes, which suggests that targeted intervention for just those subsets could be a therapeutic avenue. However, the authors did not determine why only a subset of CNS connectivity genes were most affected, and what the unifying feature(s) of those genes were.

## Behavioral and functional effects

The functional effects of hypoxia on the vertebrate nervous system are still unclear. That is, it is not apparent whether there is a ‘logic’ or purposeful adaptation associated with the changes of CNS connectivity due to hypoxia. For example, does the CNS respond to developmental hypoxia by altering connectivity such that the animal's behavior is changed, for example, to seek a more oxygen-rich environment or to consume a diet higher in iron to facilitate erythropoiesis? Alternatively, the effects of hypoxia on connectivity might simply be non-intentional outcomes of the ectopic activation of HIF1α, and not have any functional role.

In *C. elegans*, several groups have provided evidence that hypoxia alters neuronal circuitry and that the accompanying behavioral changes are adaptive. Following exposure to chronic hypoxia in adult animals, *C. elegans* will preferentially choose conditions of low oxygen and avoid hyperoxia, with altered responses to feeding (Chang and Bargmann, 2008). This effect is mediated by changes in the network of interactions between different neurons, although the authors did not evaluate specific changes in synapse or axon connections. The authors propose that this response is adaptive because the new behavior results in animals still feeding in low-oxygen conditions, whereas control animals not previously exposed to hypoxia reduced their feeding. Work from the Hobert laboratory (Pocock and Hobert, 2010) showed that hypoxia led to the activation of an alternative gustatory circuit, via a mechanism involving increased expression of 5-HT, although they also did not evaluate for changes in axon or synapse connections. They propose that this circuit is adaptive because it enhances sensory acuity in a potentially hostile (hypoxic) environment.

Whether changes in circuitry from developmental exposure to hypoxia alter the organism's behavior in an adaptive fashion in the more complex vertebrate CNS is unclear. Using the zebrafish system, the Bonkowsky group demonstrated that developmental hypoxia decreases 5-HT expression, and that changes in 5-HT levels can specifically change axon pathfinding of a group of midline axons (Xing et al., 2015). This finding suggested that levels of 5-HT, regulated through the molecular response to hypoxia, responsively alter midline axon crossing. Reduced oxygen levels result in reduced 5-HT expression and fewer axons crossing the midline, and thus act as a kind of thermostat to change connectivity. However, the study did not evaluate whether hypoxia and the change in axon pathfinding altered the behavior of either larvae or adult zebrafish.

Do humans have an adaptive response of CNS circuitry to developmental hypoxia? To our knowledge, there are no studies focusing on this question, nor are there studies using other mammals (such as mouse). However, indirect evidence suggests that a similar mechanism could exist in humans involving 5-HT and hypoxia. First, 5-HT axon projections are widespread when extensive axon pathfinding occurs in early development (Rubenstein, 1998; Lillesaar et al., 2009). Second, gestational exposure to serotonergic drugs has been linked with increased risks for NDDs (Rai et al., 2013; El Marroun et al., 2014; Gidaya et al., 2014; Harrington et al., 2013). Third, polymorphisms in genes in the 5-HT signaling pathway are associated with a range of neuropsychiatric disorders (Sutcliffe et al., 2005; Prasad et al., 2009; Kuzelova et al., 2010). Finally, early loss of 5-HT neurons or other components of 5-HT signaling leads to diffuse CNS abnormalities with a wide range of behavioral phenotypes (Daubert and Condron, 2010; van Kleef et al., 2012).

## Evidence from evolution and from normal development links hypoxia, HIF1 and connectivity

**Fig. 3.**
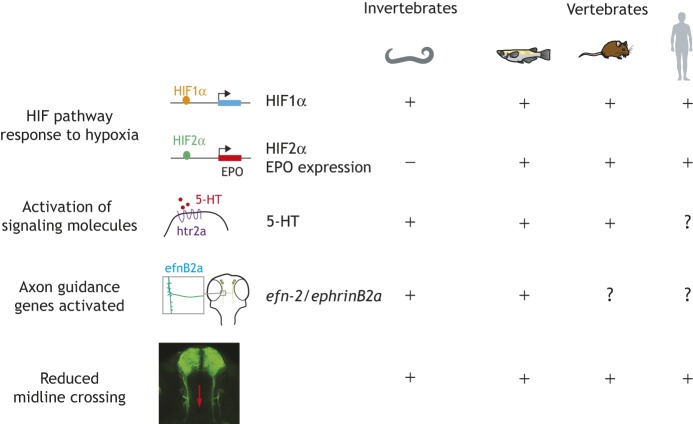
**Examples of conserved CNS connectivity responses to hypoxia across evolution, including metazoans with bilaterally symmetric nervous systems.** There are no HIF homologs in yeast or primitive metazoans. Animals shown are nematodes (*C. elegans*), fish species including zebrafish, mice and primates/humans.

A striking feature of the HIF1α response to hypoxia is how highly conserved it is across multicellular organisms, from *C. elegans* to zebrafish to mouse to human (Fig. 3). This includes changes in axon pathfinding, the signaling molecules and genes downstream of HIF1α that cause the changes in connectivity, and possibly synaptic connectivity. However, different animal species appear to have evolved different tolerances to hypoxia. For example, some aquatic species such as zebrafish can tolerate complete anoxia during development with no apparent adverse effects, although the differential responses by different species, and the mechanisms, are poorly understood (Mendelsohn et al., 2008, 2009; Cai et al., 2018).

Multicellular animal (metazoan) life first evolved in what is now considered a hypoxic environment of 2-4% oxygen levels (Canfield, 2014), and did not reach modern oxygen levels of >10% oxygen until the end of the Proterozoic era and the start of the Cambrian era 541 million years ago (Sperling et al., 2015). There do not appear to be HIF1α homologs in non-metazoan yeast (*Saccharomyces cerevisiae*) or in an early-diverging metazoan sponge (*Amphimedon queenslandica*), neither of which have bilaterally symmetric nervous systems. Interestingly, HIF2α is only present in vertebrates, and erythropoietin, which is regulated by HIF2α, first appeared during evolution in fish species (Hammarlund et al., 2018). Both PHD and HIF1 proteins are present in invertebrate animals with bilaterally symmetric nervous systems such as *Drosophila* and *C. elegans* (Rytkönen et al., 2011). Thus, all animal species examined to date with bilaterally symmetric nervous systems and conserved mechanisms of axon pathfinding and synaptogenesis use the HIF1α and PHD systems in their response to hypoxia.

An interesting consideration is how the evolution of metazoan life in the low-oxygen conditions of Earth's early history might be important for understanding CNS connectivity development. The mammalian fetus and brain develop in relatively low-oxygen conditions (Dunwoodie, 2009). Even in the adult human brain, oxygen levels range only from 0.5 to 8% (6-33 mm Hg) (Dings et al., 1998). During embryogenesis, a physiological hypoxia of ∼3% pO_2_ [partial pressure of oxygen (Box 2); 24 mm Hg] is necessary in the brain for the maturation and differentiation of neural stem cells and radial glia, and *in vitro* work has shown that lower or higher oxygen levels adversely affect those processes (Ortega et al., 2017). Thus, physiological hypoxia is an essential component of normal CNS development, further supporting a physiological and evolutionarily conserved role for HIF1α in these processes.

## Conclusions and discussion

In this Review, we have discussed the recent literature on the effects of hypoxia on the development of axon and synaptic connections in the vertebrate CNS. Hypoxia is a physiological state during some stages of CNS development, but can also cause abnormal development if it is more severe or altered in timing.

Three major areas of research deserve additional study regarding hypoxia and CNS connectivity development. The first is whether the vertebrate CNS responds to hypoxia with changes in circuitry that have adaptive effects on the organism's behavior. It is clear that hypoxia disrupts connectivity, and that these disruptions can lead to changes in behavior, including NDDs such as autism. However, it is unclear whether the hypoxia-induced connectivity changes serve to alter CNS function or behavior in some fashion that could be construed as adaptive.

The second question is, to what extent do physiological hypoxia and the HIF1α pathway regulate the normal development of CNS connectivity? While the activation of HIF1α and its downstream effectors by hypoxia can disrupt connectivity development, the evidence of physiological hypoxia during development and work on Hif1α knockout mice support a role for HIF1α during normal development. Finally, despite the evolutionary conservation of the hypoxia response pathway, the extent to which hypoxia is disrupting or altering CNS connectivity in humans, for example in premature infants, is still poorly understood.

Can the models and findings regarding hypoxia and its effects on CNS connectivity be applied clinically? The research findings described in this Review, and the conservation of genes (Howe et al., 2013) and of hypoxia mechanisms and pathways between species, support this possibility. However, clinical adoption is so far lagging. Conversely, research into the basic mechanisms of hypoxia's effects on brain connectivity is proportionately underrepresented relative to the significant human health implications. An appealing approach to encourage clinical translation would be the use of a small vertebrate organism such as zebrafish (MacRae and Peterson, 2015) to perform high-throughput compound screens to rescue the adverse effects of hypoxia. Candidate compounds could lead to new clinically relevant therapeutic avenues for treating the adverse effects of prematurity and hypoxia. We expect that continued advances in the characterization of hypoxia and HIF1α will reveal novel insights into the mechanisms of CNS connectivity development and the etiologies of NDDs.
